# Relationship between the chemical composition, textural attributes, and sensory acceptability of *Tofu* as influenced by different coagulants

**DOI:** 10.3389/fnut.2025.1724587

**Published:** 2025-12-17

**Authors:** Wasiu Awoyale, Martha Shirley Epiphaneia Williams-Ngegba, Charis Ifeoluwa Laoye, Lateef Oladimeji Sanni, Busie Maziya-Dixon

**Affiliations:** 1 Department of Food Science & Technology, Kwara State University, Malete, Kwara, Nigeria; 2 Post-Harvest Engineering, Food and Nutrition Sciences, Njala Agricultural Research Centre, Sierra Leone Agricultural Research Institute, Njala, Sierra Leone; 3 The Directorate, Nigerian Stored Product Research Institute, Ilorin, Kwara, Nigeria; 4 International Institute of Tropical Agriculture, Sierra Leone Country Office, Freetown, Sierra Leone

**Keywords:** coagulants, soymilk, *Tofu*, chemical composition, textural attributes, sensory acceptability

## Abstract

**Introduction:**

It has been established by various researchers that the type of coagulants affects the quality of *Tofu*. Still, no work has been published on how the chemical composition and textural attributes influence the sensory acceptability of *Tofu*. This study aims to assess the relationship between the chemical composition, textural attributes, and sensory acceptability of *Tofu*.

**Methods:**

Soymilk was produced from soybeans, with soymilk protein denatured by heat, and curdled using different coagulants like vinegar, lime juice, alum solution, and steeped *ogi* water to get different samples of *Tofu*. The *Tofu* samples were evaluated for chemical composition, textural attributes, and sensory acceptability using standard methods.

**Results and discussion:**

The results showed that the vinegar-coagulated *Tofu* significantly possesses the highest fat, ash, crude fiber, total carbohydrate, and total energy contents, and the steeped *Ogi* water-coagulated *Tofu* had the highest protein content. The calcium, magnesium, and zinc contents were higher in the vinegar-coagulated *Tofu*, while the sodium content was higher in the alum solution-coagulated *Tofu*. Total phenolics and total flavonoids were higher in the vinegar-coagulated *Tofu*, while the steeped *Ogi* water-coagulated *Tofu* had the highest DPPH value. The lime-coagulated *Tofu* had the lowest of all the chemical compositions. The adhesiveness, chewiness, cohesiveness, and gumminess were higher in the vinegar-coagulated *Tofu*, while the fracturability and hardness were higher in the lime juice-coagulated *Tofu*. Steeped *Ogi* water was shown to be the most effective coagulant in improving the sensory aspects of *Tofu*, followed by vinegar, providing a tasty and aesthetically beautiful product, while lime was the least popular choice. The calcium and zinc contents, total flavonoid and phenolic contents, and DPPH may have also contributed to the fracturability of the steeped *Ogi* water-coagulated *Tofu*, and the protein content may have contributed to the springiness and subsequent overall acceptability of the steeped *Ogi* water-coagulated *Tofu*.

**Conclusion:**

Therefore, steeped *Ogi* water could be used to produce quality *Tofu* that will balance the chemical composition, textural attributes and sensory acceptability.

## 1 Introduction

Soybean, a highly nutritious plant legume, contains well-balanced amino acids and desirable fatty acids. It serves as a crucial protein source for many people worldwide (1, 2). Food formulations that incorporate soy proteins into different products are being exploited by food experts and manufacturers as functional foods (3, 4). The increasing acceptance of soy foods by the general population is due to the growing recognition of the health benefits of these foods, especially among skeptics who aim to reduce their consumption of animal products (5). Besides, the medicinal nature of soybeans is extremely essential in building the body's immune system. Soybean foods have significant protection against such health challenges as heart disease, diabetes, high blood pressure, stroke, menopause, ulcers, and cancer (6, 7). An example of a value-added product from soybeans is soymilk.

Soymilk is the type of milk that is most closely related to dairy milk among non-dairy milk products (4). Soymilk has nearly the same nutritional proportions as cow's milk, including 3.5% protein, 2% fat, 2.9% carbohydrate, and 0.5% ash, and it is also rich in protein, vitamins, and minerals (8). In the dairy industry, soymilk and soybean proteins compete with dairy and milk proteins as low-cost substitutes. Beyond its health attributes, soymilk fulfills dairy milk shortages in some regions of the world (9). Generally, about 80% of the soy proteins extracted from soybeans are glycinin (11S) and β-conglycinin (7S), which can be precipitated at pH 4.5 and, as such, are called acid-precipitated proteins (10). On the other hand, some proteins remain soluble at pH 4.5 and are thus called whey soybean proteins, which make up 9%−15.3% of soybean seed protein (10). Soymilk can be transformed into another value-added product through the denaturation and coagulation of the glycinin (11S) and β-conglycinin proteins to get *Tofu* (10).

*Tofu*, which is the coagulated and insoluble part of the soymilk protein (glycinin and β-conglycinin), can be used as an inexpensive meat substitute; it is cholesterol-free, a rich source of proteins, minerals and omega-6 polyunsaturated fatty acids (11). It possesses numerous health benefits, such as its ability to lower cholesterol levels, prevent anemia and manage weight (11). It also helps in maintaining cardiovascular health and lowers the risk of cancer, anemia, osteoporosis and kidney diseases (12). In addition, it helps in lowering the levels of bad cholesterol (11). However, coagulating soymilk with or without other additives to make *Tofu* has a long history, and these products have been used by people all over the world (9). It is important to add that *Tofu* is known by different names in different regions of the world. The general and English names are bean curd, soybean curd, vegan paneer, and soy cheese. In Nigeria, *Tofu* is known as *awara, beske, wara* soya, and soya cheese (1–4). In Asia, *Tofu* is known as *doufu*, silken *tofu*, firm *tofu, yuba* and *Fu ru* (5, 7, 8, 11). But, in the context of this paper, *Tofu* will be used throughout the discussion. The yield and quality of *Tofu* are said to be determined by the quality of the soymilk used and the type of coagulants.

The uses of some coagulants exist in scientific literature, such as sunflower, pineapple, several plant preparations of *Cynara cardunculus*, steeped ogi water, lime juice, alum, vinegar, and CaCl_2_ (4, 13). These coagulants have also been reported to impart both physical and chemical properties as well as the sensory effects on the quality of the curds (4, 14). It is envisaged that the chemical composition of the *Tofu* may be affected by different coagulants, which may subsequently affect the textural attributes and sensory acceptability of the *Tofu*. However, despite the wide application of these coagulants in *Tofu* production, the relationships between the chemical composition, textural attributes, and sensory acceptability of the product as influenced by different coagulants have not been documented or published. It is therefore important to assess how different coagulants influence the relationship between the chemical composition, textural attributes, and sensory acceptability of *Tofu*.

## 2 Materials and methods

### 2.1 Materials

The quality soybean, lime, vinegar, and alum were procured from Oja-Oba Market, Ilorin, Kwara State, Nigeria. The steeped *Ogi* water was obtained from Ogi, processed at the Food Processing Laboratory, Department of Food Science and Technology, Kwara State University, Kwara State, Nigeria.

### 2.2 Production of *Tofu*

*Tofu* was produced as described by Ndife et al. (4). Soybean seeds (2 kg) were sorted manually, washed using clean water, steeped in clean water with the soybean seed water ratio of 1:3 weight/volume for 12 h, dehulled manually between palms, drained, and milled using a locally fabricated attrition mill with a soybean water ratio of 1:8 weight/volume. The milled soybeans were filtered using muslin cloth to separate the soymilk from the insoluble residue, known as okara. Then, the obtained soymilk (12 L) was divided into four portions (3 L each) to produce *Tofu*. The *Tofu* was produced by adding coagulants to the soymilk in different ratios as shown in Table 1. The coagulants used are lime juice, vinegar, alum solution, and steeped *Ogi* water. The steeped *Ogi* water was collected from *Ogi* produced from white-seeded maize grain by soaking a specific quantity of maize (5 kg) in a fixed volume of water (20 L) for 4 days. The water was decanted, and the maize grain was washed and wet milled using a locally fabricated attrition mill. The milled maize paste was mixed with clean water in a ratio of 1:3 by weight per volume, respectively. This was followed by wet sieving using a muslin cloth and allowed to sediment overnight at room temperature (30±2 °C). The supernatant (steeped *Ogi* water) was decanted into a clean bowl, kept for 3 days, and used for the experiment. The pH of the steeped *Ogi* water was measured using a pH meter (Mettler Toledo GmbH; 8606 Greifensee, Switzerland) (15) and was found to be 3.8. However, the pH of the steeped Ogi water depends on the temperature and period of fermentation, and the variety of maize. A Specific amount of the different coagulants was added to a specific quantity of milk inside a stainless-steel pot, bit by bit, on a gas cooker maintained at a low temperature as the milk started to boil to induce coagulation, without stirring. The soy curd was produced in batches, depending on the curd's formation, and collected into the muslin cloth. The curds were partially drained inside the muslin cloth, with the addition of salt (1.5 g), seasoning (1 g), and sliced fresh pepper (5 g), and pressed to expel the whey before cutting into rectangular uniform sizes.

**Table 1:** Curdling soymilk using different coagulants.

Coagulants^*^	**Amount (ml)**	**Soymilk (ml)**
Lime juice	600	3,000
Vinegar	200	3,000
Alum solution	600	3,000
Steeped *Ogi* water	1,500	3,000

^*^The lime juice was extracted from the lime fruits. The alum solution was produced by mixing the alum and distilled water in a ratio of 1:2 weight/volume. The difference in the volume of the coagulants was due to their coagulating strengths derived from a baseline study.

### 2.3 Chemical composition

#### 2.3.1 Moisture content

The moisture content of the samples was determined using the AOAC (15) method. Two grams of the samples were put in oven dishes and placed in a hot air oven (Memmert GmbH+co, Oven model D-91126 Schwabach FRG, Germany) at 100 °C until a constant weight was achieved. The weight loss due to evaporation of moisture was recorded and calculated using Equation 1.


$$\text{Moisture }(\%)=\frac{\left(W_{1}-\;W_{2}\right)}{W_{1}-\;W_{0}}\times 100$$ (1)

Where: *W*_o_ = Weight of the empty crucible; *W*_1_ = weight of the powder sample + empty crucible; *W*_2_ = weight of dried sample + empty crucible.

#### 2.3.2 Crude protein content

The determination of protein content of samples was carried out following the AOAC method (15). One gram of sample was measured, put into a 300-ml Kjeldahl tube, and hydrolysed using concentrated sulfuric acid. The KjelDigester apparatus (K-446/K-449 BUCHI) was preheated to a temperature of 420 °C, and the Kjeldahl tube was attached to the KjelDigester apparatus. The scrubber unit was turned on, and the digestion process ran for 1 h. Thereafter, the tube was put into the distillation unit. The protein content was calculated as shown in Equation 2.


$$\begin{array}{l} \text{Protein content }\left(\text{\%}\right)=\frac{\left(V_{p}-\;V_{b}\right)\;\times \;1.4007\;\times \;Fk}{Same\;weight\;\left(g\right)}\;\times \;100\% \end{array}$$ (2)

Where:

*V*_p_ = Volume of 0.2 N HCl required for sample titration (ml)

*V*_b_ = Volume of 0.2 N HCl required for blank titration (ml)

N = Normality of 0.2 N HCl solution

Fk = Protein conversion factor

#### 2.3.3 Crude fat content

The fat content of the samples was determined by Soxhlet extraction as described by AOAC (15). The samples will first be treated through acid hydrolysis to remove impurities. The two grams of the treated samples will then be put into an extractor thimble, sealed with cotton wool, and placed into the Soxhlet apparatus. Equation 3 was used for calculating the fat content of the samples.


$$\begin{array}{l} \text{Crude fat }\left(\text{\%}\right)=\frac{\left(C\;-\;A\right)}{B}\times 100\% \end{array}$$ (3)

Where:

*A* = weight of the empty fat flask (g)

*B* = weight of test portion (g)

*C* = Fixed weight of fat flask + test portion after heating (g)

#### 2.3.4 Crude fiber content

Using 2 g (*W*_3_) of the sample, the crude fiber content was determined following the AOAC (15) method. The flask was filled with approximately 200 ml of 1.25% (v/v) sulfuric acid, placed on a hot plate, and allowed to boil for 30 min. Filter paper was used to filter the content, and 50–70 ml of distilled water was used to remove any remaining residue. The rinsed residue was removed, 200 ml of 1.25% (w/v) NaOH was added, and the mixture was heated for 30 min. The content was then filtered as previously stated, and the residue that was left over was then cleaned with distilled water before being filtered once more with filter paper. After being moved to an ashing dish, the residue was dried at 130 °C for 2 h, cooled in a desiccator, and weighed (*W*_1_). After 30 min, the mixture was ashed at 550 °C in a muffle furnace (VULCANTM furnace type 3-1750), cooled and weighed again (*W*_2_). Equation 4 was used to calculate the crude fiber content.


$$\begin{array}{l} Crude\;fiber\;content\;\left(\%\right)=\frac{W_{1}-W_{2}}{W_{3}}\times 100 \end{array}$$ (4)

*W*_1_ = mass of crucible with dried residue (g)

*W*_2_ = Mass of crucible with the ash (g)

*W*_3_ =Weight of sample

#### 2.3.5 Ash content

A muffle furnace (Ney Vulvan TM furnace type 3-1750, United States) was used to measure the ash content of the samples according to the established AOAC (15) method. Two grams (*W*_3_) of the sample were weighed into an ashing crucible that had already been weighed (*W*_2_), and the samples were then placed in the muffle furnace chambers at 700 °C for 3 h, during which time they were reduced to ashes. After being removed, the crucible was cooled in a desiccator and weighed (*W*_1_). Equation 5 shows how the ash content was calculated as a proportion of the weight of the original sample.


$$\begin{array}{l} Ash\;content\;\left(\%\right)=\frac{W_{1}-W_{2}}{W_{3}}\times 100 \end{array}$$ (5)

*W*_1_ = Mass of crucible + Ash

*W*_2_ = Mass of empty crucible (g)

*W*_3_ = Weight of sample

#### 2.3.6 Carbohydrate content

The carbohydrate content of the samples was determined as described by AOAC (15). The carbohydrate content of the samples was determined by subtracting the difference from the sum of moisture, protein, fat, crude fiber, and ash contents from 100, as shown in Equation 6.


$$\begin{array}{l} \%\text{Carbohydrate}=100-(\%\;moisture+\%\;ash+\\ \;\%\;protein+\%\;lipids+\%\;crude\;fibre) \end{array}$$ (6)

#### 2.3.7 Total energy value

The energy value of the samples was calculated using the mathematical expression shown in Equation 7.


$$\begin{array}{l} \text{Energy value}=[(\%\text{ Fat}\times \text{9})\;+(\%\text{ Protein}\times \text{ 4})\;+\\ \;(\%\text{ Carbohydrate }\times \text{4})] \end{array}$$ (7)

#### 2.3.8 Mineral composition

In accordance with AOAC (15), an atomic absorption spectrophotometer (Buck Scientific model 210 VGP) was used to determine the calcium, magnesium, zinc, and sodium concentrations of the samples. Three hours at 600 °C in a carbonite muffle furnace were used to incinerate 2 g of the samples. Exactly 10 ml of 6 N HCl was added to the ash sample, which was then put in a water bath and cooked for 10 min. The samples were removed and put into a 100 ml volumetric flask. Deionised water was used to dilute the filter paper until it was 100 ml in volume. The digested sample was divided into 10 ml pieces, placed in a sample container, and aspirated into an atomic absorption spectrophotometer, and the amount of calcium, magnesium, zinc, and sodium in parts per million (ppm) was recorded.

#### 2.3.9 Preparation of an extract from *Tofu* samples for antioxidant analysis

About 20 g of *Tofu* from each coagulant was soaked in 200 ml of methanol (80%), and the solution was continuously stirred at 200 rpm for a period of 120 h using an orbital shaker (Thermo Fisher Scientific, MaxQ 6000, United States) at room temperature (30±2 °C), followed by the separation of the mixture through a filtration technique using Whatman filter paper No. 4. These filtrates were concentrated using a vacuum rotary evaporator at 45 °C. About 5 ml of every extract was stored at 4 °C to perform further analysis (16).

##### 2.3.9.1 Total phenolic content

The total phenolic content of the samples was determined as described by Hussain et al. (16). Accurately, 0.5 ml of each extract, 2.5 ml of Folin-Ciocalteu reagent (100 g/k), and 2 ml of NaCO_3_ (75 g/kg) were added into a tube and mixed well. This mixture was given a stay time of 15 min at 50 °C. Then the absorbance was measured at 760 nm using a spectrophotometer (Hitachi U-2001, Hitachi Instruments Inc., Tokyo, Japan), against a methanol-water solution (80:20, v:v) as a blank solution. The results for total phenolic contents of samples were expressed as mg/100 g sample of gallic acid equivalent (GAE).

##### 2.3.9.2 Total flavonoid content

The total flavonoid content of the samples was determined as described by Hussain et al. (16). Extracts of each sample (1 ml) and 0.3 ml NaNO_3_ (50 g/kg) were mixed homogeneously in a test tube, followed by the addition of 0.3 ml Al (NO_3_)_3_ (100 g/kg) and 4 ml NaOH (50 g/kg). A stay time of 15 min was given to the solution, and then the absorbance was measured at 510 nm using a spectrophotometer, against a methanol-water solution (80:20, v:v) as a blank solution. The total flavonoid contents of the samples were then expressed as mg/100 g sample of Catechin equivalent (CE).

##### 2.3.9.3 DPPH free radical scavenging activity

The free radical scavenging activity of the samples was determined as described by Hussain et al. (16). An amount of 0.01 g of 2,2-diphenyl-1-picrylhydrazyl (DPPH) was poured into a 25 ml flask containing solvent (80:20 methanol/water; v/v). A calibration curve of ascorbic acid was also established. An amount of 100 μl from each type of extract was collected in microplates, then an amount of 2.0 ml solvent and 250 μl DPPH reagent were added. These microplates were shaken and kept in darkness at ambient temperature for a period of 20 min. Reduction in DPPH absorbance by spectrophotometer, at 520 nm, was witnessed every 5 min intervals unless this absorbance was stabilized after a period of 30 min. Methanol played its role as a blank solution, while the DPPH solution with no test samples acted as a control. The results were expressed as mg of ascorbic acid equivalent (AAE)/100 g sample in a reaction time of 30 min.

### 2.4 Texture profile analysis

A TA-XTPlus-Stable Microsystems, United Kingdom, texture analyser equipped with a 50 kg load cell, a probe diameter of 100 mm, deflection of 50%, speed of 102 mm/min, Preload of 20 mm and a thickness of the cutting ring of 20 mm, was utilized for the instrumental texture profile analysis of the *Tofu*. *Tofu* adhesiveness, chewiness, cohesiveness, fracturability, gumminess, hardness, and springiness are the texture characteristics derived from the analyser. During the evaluation, the *Tofu* samples were kept in a cooler at 28 °C−30 °C (17).

### 2.5 Sensory evaluation

The sensory properties of the *Tofu* samples were evaluated using a well-structured questionnaire with 20 trained panelists selected among the students of the Kwara State University, Malete, Kwara State. Each panelist was presented with four different coded samples of *Tofu* produced from different coagulants. Sensory evaluation was carried out by scoring each sample on a 9-point hedonic scale (where 9 = extremely liked and 1 = extremely disliked). The sensory attributes evaluated on the *Tofu* are taste, texture, color, appearance, aroma, and overall acceptability. The Kwara State University Ethical Committee in Malete, Kwara State, issued ethical permission to conduct this study following the Helsinki Declaration of 1975 on human experimentation.

### 2.6 Statistical analysis

All the data were replicated except for the sensory evaluation, which was done using 20 panelists. Using the Statistical Package for Social Sciences (SPSS version 21) software, the obtained data were subjected to analysis of variance (ANOVA) and *post-hoc* tests, and the means were separated with Duncan's multiple range test at a probability level of *p* < 0.05. The Principal Component Analysis was performed using the free version of XLSTAT software, 2023.

## 3 Results and discussion

### 3.1 Chemical composition of *Tofu* from different coagulants

The chemical composition of *Tofu* from different coagulants is shown in Table 2. The mean values are moisture 47.70%, protein 22.40%, fat 5.78%, ash 1.83%, crude fiber 0.51%, total carbohydrate 21.80%, and total energy 228.90 kcal/g. Significant differences (*p* < 0.05) exist in all the proximate compositions of the samples.

**Table 2:** Chemical composition of *Tofu* from different coagulants.

***Tofu* from different coagulants**	**Moisture content (%)**	**Protein content (%)**	**Fat content (%)**	**Ash content (%)**	**Crude fiber content (%)**	**Total carbohydrate content (%)**	**Total energy content (kcal/g)**
Steeped *Ogi* water	47.10 ± 0.17^c^	25.90 ± 0.04^a^	5.74 ± 0.02^c^	2.01 ± 0.06^a^	0.54 ± 0.02^a^	18.7 ± 0.04^d^	230.20 ± 0.50^b^
Alum solution	49.50 ± 0.11^a^	21.50 ± 0.01^c^	5.43 ± 0.04^d^	1.60 ± 0.04^c^	0.48 ± 0.01^b^	21.6 ± 0.08^b^	220.90 ± 0.69^d^
Lime juice	48.30 ± 0.03^b^	23.40 ± 0.04^b^	5.92 ± 0.02^b^	1.73 ± 0.03^b^	0.49 ± 0.01^b^	20.2 ± 0.05^c^	227.40 ± 0.16^c^
Vinegar	45.80 ± 0.02^d^	18.70 ± 0.02^d^	6.03 ± 0.04^a^	1.98 ± 0.04^a^	0.54 ± 0.01^a^	26.9 ± 0.10^a^	236.90 ± 0.04^a^
Mean	47.70	22.40	5.78	1.83	0.51	21.80	228.90
*p* level	^***^	^***^	^***^	^**^	^*^	^***^	^***^

Values are mean ± standard deviation of duplicate determinations. ^*^p < 0.05, ^**^p < 0.01, ^***^p < 0.001. Data with different superscripts across the same column are significantly different (*p* < 0.05).

The moisture content of the *Tofu* samples ranged from 45.80 to 49.50%. The moisture content was higher in the alum solution*-*coagulated *Tofu* and lower in that of the vinegar. The high moisture content in alum solution-coagulated *Tofu* may be attributed to its ionic strength and ability to form finer protein networks that entrap more water compared to that of the vinegar-coagulated *Tofu* (18). The result of this study contradicts some earlier assumptions that natural coagulants like *steeped Ogi water* inherently yield softer curds with higher moisture. Instead, it appears that the alum-induced network retained more free water, possibly due to less syneresis or firmer gel formation (7). The moisture content observed in this study is higher than 20.40%−25.33% reported for the moisture contents of lime, steeped water dried and fried *Tofu* reported by Raji et al. (13), and 20.04%−28.16% for the moisture contents of *Tofu* coagulated with corn steep liquor and tamarind studied by Ojochogu et al. (19). However, the moisture content of the *Tofu* in this study is lower than the values reported for *Tofu* coagulated with different coagulants by Shi et al. (20) (57.70%−76.30%), and Obatolu (21) (70.60%−79.90%). Variation in moisture contents of the *Tofu* samples could be attributed to differences in the gel network structures of the *Tofu* particles and the influence of different anions and ionic strengths on the water holding capacity of soybean protein gel (20).

The protein content of the *Tofu* samples varied significantly (*p* < 0.05) from 18.70 to 25.90%. The highest protein content was found in the steeped *Ogi* water*-*coagulated *Tofu*, while the lowest was found in the vinegar-coagulated *Tofu*. The significant variation in the protein content of the samples may be attributed to protein-binding capacities and pH environments induced by the different coagulants (22). Steeped *ogi* water, being mildly acidic with natural lactic fermentation components, may enhance protein coagulation and retention better than acetic acid from vinegar (22). The variation in the protein contents of the *Tofu* samples may be attributed to the distinct mechanisms by which different coagulants induce protein precipitation (22). Steeped *Ogi* water, rich in organic acids from fermented cereal substrates, likely promotes an environment near the isoelectric point of soy proteins (around pH 4.5), thus enhancing protein aggregation and curd formation (23). In contrast, the low protein content of the vinegar-coagulated *tofu* may be attributed to the acidic nature of vinegar, which may rapidly denature soy proteins, resulting in a less dense, less compact protein gel with more water and potentially fewer aggregated proteins to trap them (23). The values obtained in this study are higher than 4.57%−7.18% reported for crude protein contents of differently processed *Tofu* (24), and 14.37%−17.56% for protein contents of *Tofu* coagulated with alum and lime (25). A lower range of protein content (7.70%−18.50%) compared to that of this study was also reported by Shi et al. (20) from different coagulants. However, the protein content (40.00%−41.40%) of *Tofu* coagulated with lime, alum, and *steeped Ogi water* reported by James et al. (7), and *Tofu* from different coagulants (54.20%−58.20%) reported by Obatolu (21) was higher than that of this study. The fat content of the *Tofu* samples ranged from 5.43 to 6.03%. The vinegar-coagulated *Tofu* had the highest fat content, while the lowest value was recorded in alum-coagulated *Tofu* (*p* < 0.05). The difference in the fat content might reflect how each coagulant interacts with the soymilk during curd formation, especially in terms of how tightly or loosely the curd holds together and how much liquid is expelled (22). The findings of the current work are in tandem with 5.53%−7.56% reported for fat contents of alum, potassium aluminum sulfate, tamarind fruit pulp extract, *Moringa oleifera*, and Lime juice-coagulated *Tofu* (4) but lower than 19.90%−21.70% for fat contents of *Tofu* coagulated with steeped *Ogi* water, alum, *Calotropis procera*, and calcium chloride (26) and 31.10%−34.40% for fat contents of lime, steeped *Ogi* water dried and fried *Tofu* reported by Raji et al. (13). The variation in fat contents of the *Tofu* samples could be attributed to the ability of the different coagulants to allow the release of fat during processing (21).

The steeped *ogi* water-coagulated *Tofu* (2.01%) had the highest ash content, while the alum solution-coagulated *Tofu* had the lowest (1.60%) (*p* < 0.05). However, the ash content of the vinegar-coagulated *Tofu* was not significantly different (*p* > 0.05) from that of the steeped *ogi* water-coagulated *Tofu*. The relatively higher ash content observed in the steeped *ogi* water-coagulated *Tofu* suggests a better retention of minerals, which may be attributed to the fact that steeped *ogi* water is a fermented cereal extract known to contain essential minerals such as calcium, magnesium, potassium, and phosphorus (27, 28). Additionally, the interaction between the steeped *ogi* water coagulant and the soymilk matrix during curdling could play a role in trapping or preserving more of the inherent minerals in the protein network, hence the higher ash content (22). On the contrary, the ash content of the *Tofu* of this study is lower than 2.77%−3.40% reported for ash contents of lime, steeped water dried and fried *Tofu* by Raji et al. (13) and 4.40%−6.30% for ash contents of CaSO_4_, Citrus lemon, *Hibiscus sabdariffa, Tamarindus indica* and steeped *Ogi* water coagulated *Tofu* by Ndatsu et al. (29), as well as the ash content of 5.20%−7.90% reported by Obatolu (21).

The crude fiber content was higher in the steeped *ogi* water and vinegar-coagulated *Tofu* (0.54%) and lower in that of the alum-coagulated *Tofu* (0.48%). Significant difference (*p* > 0.05) was not observed in the crude fiber content of the alum and lime juice-coagulated *Tofu*. The findings of the current study are in tandem with 0.45%−0.50% reported by Ibironke and Alakija (30) for soy cheese from vegetable protein using different coagulants, but lower than 2.0%−3.0% for crude fiber contents of lime, steeped water dried and fried *Tofu* reported by Raji et al. (13) and 10.01%−12.17% for the crude fiber contents of cheese coagulated with steeped ogi water and tamarind reported by Ojochogu et al. (19).

The total carbohydrate content of the *Tofu* ranged from 18.70 to 26.90%, with the steep *Ogi* water-coagulated *Tofu* having the lowest value, and the vinegar-coagulated *Tofu* having the highest. The trend noticed in the *Tofu* sample, where an increase in protein content comes with a drop in carbohydrate content, is quite normal for soy-based foods. This usually happens because some coagulants help the protein clump together more tightly, forming a firmer curd that traps more protein. But in doing so, it pushes out other nutrients like carbohydrates, especially the ones that dissolve easily in water. So, the more effective the coagulant is at binding protein, the less room there is for carbohydrates to remain in the final product (31). Similar results have been reported by Ezeama and Dobson (32) for carbohydrate contents (17%−26%) of *tofu* treated with different coagulants and 16.32%−17.10% for *Tofu* coagulated using different coagulants (33).

The vinegar-coagulated *Tofu* had the highest total energy content (236.90 kcal/g), while the alum solution-coagulated *Tofu* had the lowest (220.90 kcal/g). These differences in energy values among the *Tofu* samples can largely be attributed to the combined influence of their fat and carbohydrate contents (22). Both nutrients are major contributors to the total caloric value of a food product—fat provides about 9 kcal/g, while carbohydrates supply around 4 kcal/g. Therefore, even slight variations in either component can significantly affect the overall energy density of the samples. For instance, the vinegar-coagulated *Tofu* had the highest fat content, and it also recorded the highest carbohydrate value, which may explain why it had the greatest energy value among the samples. The results of this study are lower than 308.57–329.88 kcal/g for *Tofu* coagulated using different coagulants (33).

### 3.2 Mineral composition and antioxidant properties of *Tofu* from different coagulants

The mineral composition and antioxidant properties of *Tofu* samples from different coagulants are shown in Table 3. The mean values of the mineral composition are calcium 107.40 mg/100 g, magnesium 61.80 mg/100 g, zinc 3.04 mg/100 g, and sodium 8.73 mg/100 g. The mean values of the antioxidant properties are total phenolics 0.81 mg GAE/100 g, total flavonoids 37.30 mg QE/g, and 2,2-diphenyl-1-picrylhydrazyl (DPPH) 60.90 mg AAE/g. Significant differences (*p* < 0.05) exist in the mineral composition and antioxidant properties of all the *Tofu* samples. These variations reflect the influence of the different coagulants on the mineral composition and antioxidant properties in the *Tofu* samples.

**Table 3:** Mineral composition and antioxidant properties of *Tofu* from different coagulants.

***Tofu* from different coagulants**	**Minerals composition**				**Antioxidant properties**		
	**Calcium (mg/100 g)**	**Magnesium (mg/100 g)**	**Zinc (mg/100 g)**	**Sodium (mg/100 g)**	**Total phenolics (mg GAE/100 g)**	**Total flavonoid (mg QE/g)**	**DPPH (mg AAE/g)**
Steeped Ogi water	112.50 ± 0.09^b^	62.90 ± 0.03^b^	3.13 ± 0.03^b^	8.53 ± 0.03^c^	0.85 ± 0.01^b^	38.30 ± 0.18^b^	68.50 ± 0.09^a^
Alum solution	94.30 ± 0.01^d^	58.70 ± 0.10^d^	2.78 ± 0.03^d^	9.20 ± 0.02^a^	0.71 ± 0.01^d^	33.90 ± 0.23^b^	51.30 ± 0.09^d^
Lime juice	102.10 ± 0.03^c^	60.00 ± 0.03^c^	2.92 ± 0.01^c^	8.80 ± 0.05^b^	0.78 ± 0.01^c^	36.30 ± 0.18^c^	63.50 ± 0.08^b^
Vinegar	120.60 ± 0.04^a^	65.30 ± 0.04^a^	3.31 ± 0.02^a^	8.40 ± 0.01^d^	0.90 ± 0.01^a^	40.50 ± 0.21^a^	60.20 ± 0.11^c^
Mean	107.40	61.80	3.04	8.73	0.81	37.30	60.90
*p* level	^*^	^*^	^*^	^*^	^*^	^*^	^*^

Values are mean ± standard deviation of duplicate determinations. ^*^p < 0.05. Data with different superscripts within the same column are significantly different (p < 0.05).

The alum-coagulated *Tofu* had the lowest calcium content (94.3 mg/100 g), while the vinegar-coagulated *Tofu* had the highest (120.6 mg/100 g) (*p* < 0.05). The elevated calcium level in the vinegar-coagulated sample likely results from the vinegar's acidic nature (low pH value), which probably facilitated the dissolution and incorporation of more calcium ions during the curdling process (32, 34). The result of this study was higher compared with the calcium contents of *Tofu* from different coagulants reported by Ndife et al. (4) (41.03–58.01 mg/100 g), and Ezeama and Dobson (32) (3.25–11.03 mg/100 g). From a nutritional perspective, calcium is crucial for bone growth, nerve activity, and muscle function. Therefore, *Tofu* made with vinegar could provide better calcium-related health benefits, particularly in areas where dairy sources are either limited or expensive (32).

The *Tofu* samples' magnesium contents varied significantly from 30.8 to 54.3 mg/100 g. The highest magnesium content was observed in the vinegar-coagulated *Tofu* (65.3 mg/100 g), which could be attributed to vinegar's acidic properties, which may have improved mineral retention during the coagulation process (34). Contrarily, the alum-coagulated *Tofu* had the least magnesium content (58.7 mg/100 g). The result of this study is within the range of 35.10–60.96 mg/100 g reported for magnesium contents of choco-*Tofu* reported by Ajewole et al. (35) and higher compared to that of 20.33–26.20 mg/100 g reported for lime, steeped water dried and fried *Tofu* reported by Raji et al. (13). The results confirm that coagulants, such as vinegar, may be more efficient in improving the magnesium content of *Tofu*, which is essential for bone health, enzyme activity, and neuromuscular function.

The zinc content of the *Tofu* samples showed significant differences (*p* < 0.05) across the different coagulants, ranging from 2.78 mg/100 g in the alum-coagulated *Tofu* to 3.31 mg/100 g in the vinegar-coagulated *Tofu*. The high zinc content in the vinegar-coagulated *Tofu* indicates that vinegar may help in providing better zinc retention during curd formation (34). The low zinc content of the alum-coagulated *Tofu* may be attributed to the presence of aluminum ions, which may competitively inhibit zinc absorption or reduce its solubility (34). This implies that the vinegar-coagulated *Tofu* may contribute to the zinc needed for DNA synthesis, cell division, and immunological response in the human body (36). The zinc content of this study is higher than 0.08–0.76 mg/ml reported for soymilk, *nunu* and *Tofu* from Abuja and Keffi metropolis by Okpara et al. (37).

The sodium content of the *Tofu* samples varied significantly (*p* < 0.05) depending on the type of coagulant used, with values ranging from 8.40 mg/100 g in the vinegar-coagulated *Tofu* to 9.20 mg/100 g in the alum-coagulated *Tofu*. The ionic characteristics of alum, which include sodium and aluminum sulfate components, may have contributed to the *Tofu* residual sodium content (34). The sodium content of this study is lower compared to those of previous researchers; Okpara et al. (37) reported 13.12–151.0 mg/ml for soymilk, *nunu* and *Tofu* from Abuja and Keffi metropolis, while Omotosho et al. (26) reported 17.0–45.10 mg/100 g for sodium contents of *Tofu* coagulated with steeped *Ogi* water, alum, *Calotropis procera* and calcium chloride. The alum-coagulated *Tofu* may contribute to the sodium needed to participate in muscular contraction, fluid and electrolyte homeostasis, and nerve impulse transmission (38), because of its high sodium content.

### 3.3 Antioxidant properties of *Tofu* from different coagulants

Table 3 shows the antioxidant properties of *Tofu* from different coagulants. The mean values for total phenolic content, total flavonoid content, and DPPH scavenging activity were 0.81 mg GAE/100 g, 37.30 mg QE/g, and 60.90 mg AAE/g, respectively. The *Tofu* samples showed significant variations (*p* < 0.05) in all antioxidant properties.

The total phenolic contents of the samples varied from 0.71 mg GAE/100 g in alum-coagulated *Tofu* to 0.90 mg GAE/100 g in vinegar-coagulated *Tofu*. The high phenolic content of the vinegar-coagulated *Tofu* may be attributed to the fact that organic acids can better maintain or enhance phenolic chemicals during coagulation (39). The action of vinegar may also help in reducing enzymatic degradation of phenolic compounds in the vinegar-coagulated *Tofu* due to its ability to lower pH rapidly, potentially inactivating polyphenol oxidase enzymes that are known to catalyse oxidative degradation of phenolics (40). Aside from vinegar, the observation in this study for steeped *Ogi* water-coagulated *Tofu* aligns with the findings of Oboh et al. (41), who reported that *Tofu* produced using steeped *Ogi* water had significantly higher total phenol content, better reducing power, and enhanced DPPH free radical scavenging activity compared to *Tofu* made with alum or calcium salt coagulants. Oboh et al. (41) findings suggest that fermented acid-based coagulants help preserve or release polyphenolic compounds more effectively than inorganic salts, which may impair extraction or induce degradation through metal-ion complexation. The results of this study are lower than 300 mg/g reported for total phenolic contents of soy milk fermented with *Lactobacillus paracasei* by Rani and Pradeep (42) and 37.66–123.3 mg TAE/100 g for total phenolics of *Tofu* studied by Bithi et al. (43). Phenolic compounds are powerful antioxidants; due to hydroxyl groups, they act as free radical terminators. Their bioactivities may be related to their abilities to chelate metals, inhibit lipoxygenase and scavenge free radicals (42).

The vinegar-coagulated *Tofu* exhibited the highest flavonoid content (40.5 mg QE/g), followed by *Tofu* from steeped *ogi* water (38.7 mg QE/g), lime (36.1 mg QE/g), and alum (33.9 mg QE/g), which had the least value. This pattern suggests that the type of coagulant used to curdle soymilk into *Tofu* has a significant impact on the potential release of flavonoids. Flavonoids, being sensitive to processing conditions such as pH, temperature, and ionic strength, are often better preserved in environments that do not involve harsh coagulating mechanisms (44, 45). The relatively high flavonoid level in vinegar-treated *Tofu* may be due to the mildly acidic conditions favoring the stability and extractability of flavonoids (46), which is in line with the findings of Vital et al. (47) for flavonoids (32.7 mg QE/g) of soy *okara*. Contrarily, stronger ionic interactions that bind flavonoid molecules or encourage oxidative stress during coagulation may have caused alum, as a salt-based coagulant, to contribute to the breakdown or decreased extractability of flavonoids (48, 49).

The 2,2-diphenyl-1-picrylhydrazyl (DPPH) radical scavenging activity of the *Tofu* samples indicated significant variation (*p* < 0.05) based on the nature of the coagulant used. The DPPH values of the samples varied from 51.3 mg AAE/g in alum-coagulated *Tofu* to 68.5 mg AAE/g in steeped *ogi* water-coagulated *Tofu*. The high DPPH value of the steeped ogi water-coagulated *Tofu* may be attributed to the inherent bioactive composition of the fermented cereal liquor (50), which may have contributed synergistic antioxidant properties to the *Tofu*. Also, the variation in the DPPH value of the steeped ogi water-coagulated *Tofu* compared to that of the other coagulants implies that total antioxidant capacity may be influenced by additional reducing agents or synergistic phytochemicals that are naturally present in the steeped *ogi* water (50, 51), rather than just phenolic or flavonoid concentrations. The results of the current study are within the range 43.01–138.73 mg AAE/g reported for DPPH of *Tofu* by Bithi et al. (43) and 18.44–86.21 mg AAE/g DPPH radical scavenging activity reported by Azarashlkan et al. (52).

### 3.4 Instrumental texture attributes of *Tofu* from different coagulants

The instrumental texture profile results of *Tofu* samples from different coagulants are presented in Table 4. The mean values of the textural attributes of the *Tofu* samples are adhesiveness −37.1 N.s, chewiness 8.34 N, cohesiveness 0.49, fracturability 16.03 N, gumminess 7.74 N, hardness 16.03 Nand springiness 1.07. All the textural attributes of the *Tofu* samples were significantly different (*p* < 0.05) except the springiness, which was not significantly different (*p* > 0.05).

**Table 4:** Instrumental texture attributes of *Tofu* produced from the blends of different coagulants.

***Tofu* from different coagulants**	**Adhesiveness (N.s)**	**Chewiness (N)**	**Cohesiveness**	**Fracturability (N)**	**Gumminess (N)**	**Hardness (N)**	**Springiness**
Steeped *ogi* water	−68.70 ± 5.95^b^	7.63 ± 0.56^b^	0.48 ± 0.03^b^	14.70 ± 0.09^c^	7.01 ± 0.47^b^	14.70 ± 0.08^c^	1.08 ± 0.01^a^
Alum solution	0.00 ± 0.00^a^	7.99 ± 0.72^b^	0.51 ± 0.02^a^	14.60 ± 1.41^c^	7.39 ± 0.63^b^	14.60 ± 1.41^c^	1.08 ± 0.01^a^
Lime juice	−79.60 ± 1.75^c^	8.40 ± 0.21^b^	0.42 ± 0.003^c^	18.60 ± 0.45^a^	7.82 ± 0.21^b^	18.60 ± 0.45^a^	1.07 ± 0.01^a^
Vinegar	0.00 ± 0.00^a^	9.35 ± 0.29^a^	0.54 ± 0.01^a^	16.20 ± 0.33^b^	8.71 ± 0.25^a^	16.20 ± 0.33^b^	1.07 ± 0.03^a^
Mean	−37.1	8.34	0.49	16.03	7.74	16.03	1.07
*p* level	^***^	^*^	^**^	^**^	^***^	^**^	NS

Values are the mean ±standard deviation of duplicate determinations. Data with different superscripts within the same column are significantly different (p < 0.05).

NS, not significant.

^*^p < 0.05, ^**^p < 0.01, ^***^p < 0.001.

Adhesiveness, expressed as the negative force area for the first bite or the work necessary to pull the compressing plunger away from the sample (53), ranged from −79.6 to 0.00 N.s, with the lime-coagulated *Tofu* having −79.60 N.s and the alum- and vinegar-coagulated *Tofu* having 0.00 N.s. Mouthfeel may be impacted by a sticky texture, demonstrated by negative values of the adhesiveness (53). The adhesiveness of the alum and vinegar-coagulated *Tofu* is comparable to the adhesiveness of quarg *Tofu* studied by Singh et al. (54), as well as that of the *Tofu* formulated using legume flours and avocado pulp reported by Palatzidi et al. (55). Chewiness, the energy required to masticate solid food until it is ready for swallowing (56), showed significant differences among the samples (*p* < 0.05). The chewiness values ranged from 7.63–9.35 N.s, with vinegar-coagulated *Tofu* having the highest, steeped *ogi* water-coagulated *Tofu* the lowest.

The chewiness values of this study are higher than 1.11–1.67 N for the chewiness of *Tofu* (57) but lower than 348.07–547.54 N for the chewiness of Ezine *Tofu* (58). Cohesiveness, calculated as the ratio of the positive force area during the second compression portion to the positive area during the first compression, is known as the extent to which a material can be deformed before it ruptures (53). The cohesiveness of the *Tofu* samples was higher in the vinegar-coagulated *Tofu* (0.54) and lower in that of the lime-coagulated *Tofu* (0.42). However, there is no significant difference (*p* > 0.05) in the cohesiveness of the vinegar- and alum-coagulated *Tofu*. This implies that the vinegar- and alum-coagulated *Tofu* may be more resistant to disintegration, compared to the lime-coagulated *Tofu* with low cohesiveness. Also, the high cohesiveness of the vinegar- and alum-coagulated *Tofu* suggests that when exposed to mechanical forces like cutting or chewing, they are less likely to crumble or break apart. These qualities make them perfect for customers who like a hard, elastic bite, like that of well-pressed *Tofu* (57). The presence of a more tightly connected or cross-linked protein network may also be indicated by the increased cohesiveness shown, particularly in the vinegar-coagulated sample (57). The cohesiveness of the *Tofu* of this study is within the range of 0.43–0.66 reported for the cohesiveness of Aro *Tofu* (59) and 0.07–0.47 for the cohesiveness of *Tofu* (60).

Fracturability of food samples, originally regarded as brittleness, is the force at the first significant break in the curve on the force-time graph. It appears as a sudden change in direction due to food samples' first point fracture or cracking or crumbling or fragmenting of structure into pieces (61). The lime-coagulated *Tofu* had the highest fracturability (18.60 N), and the alum-coagulated To*fu* had the lowest fracturability (14.60 N). There is no significant difference (*p* > 0.05) in the fracturability of the alum- and steeped ogi water-coagulated *Tofu*. However, the findings of Paula and Conti-Silva (62) for the fracturability of extruded snacks (3.70–6.70 N) are lower than the results of the current study.

Gumminess is the energy needed to break down a semi-solid food into a swallowable form. It is a secondary textural metric that is obtained by multiplying cohesion by hardness (61). The gumminess of the *Tofu* samples followed a similar trend to chewiness, varying from 7.01–8.71 N, with vinegar-coagulated *Tofu* having the highest and the steeped *ogi* water-coagulated *Tofu* having the least. Because acetic acid facilitates more effective coagulation kinetics, the vinegar-treated *Tofu* increased gumminess, indicating a denser protein matrix with stronger internal connections (61). As a result, the structure becomes more cohesive and stronger, preventing it from breaking down during chewing and providing a longer-lasting chew. In meat or *Tofu*, where customers prefer a more solid and chewy mouthfeel, such a feature may be desired (63). The gumminess recorded in this study is lower than 348.07–593.75 N for gumminess of ezine *Tofu* (58), but higher than the 2.23–2.58 N for *Tofu* (57).

Hardness, which is the peak force during the first compression and directly relates to chewiness and gumminess (17), varied significantly (*p* < 0.05) from 14.60 to 18.60 N. The lime-coagulated *Tofu* is the hardest, and the alum-coagulated *Tofu* has the least hardness. However, no significant difference (*p* > 0.05) was observed in the hardness of the alum- and steeped *ogi* water-coagulated *Tofu*. The hardness values of the *Tofu* samples in this study are higher than those (2.5–10 N) reported by Ma et al. (64), suggesting a harder texture. However, the findings of González et al. (59) for hardness 7.259–15.185 Nare in tandem with the results of the current study. The degree to which a product physically recovers from deformation during the initial compression is known as its springiness (56). All *Tofu* samples exhibited similar springiness values (*p* > 0.05). This uniformity showed that the coagulant type does not influence the elastic recovery of the *Tofu*.

### 3.5 Sensory properties of *Tofu* from different coagulants

The sensory attributes of *Tofu* samples from different coagulants are shown in Table 5. The attributes assessed include color, taste, appearance, aroma, texture, and overall acceptability. Statistically significant differences (*p* < 0.05) were observed across all the sensory attributes, indicating that the choice of coagulant may play a critical role in determining the sensory quality of *Tofu*. On average, all the sensory attributes of the *Tofu* samples from different coagulants were moderately liked.

**Table 5:** Sensory attributes of *tofu* from different coagulants.

***Tofu* from different coagulants**	**Color**	**Taste**	**Appearance**	**Aroma**	**Texture**	**Overall acceptability**
Steeped ogi water	7.67 ± 0.62^a^	7.73 ± 0.46^a^	7.33 ± 0.82^a^	7.67 ± 0.90^a^	8.07 ± 0.79^a^	7.87 ± 0.99^a^
Alum solution	7.00 ± 0.76^a^	6.80 ± 0.86^b^	6.60 ± 1.12^b^	6.73 ± 1.03^b^	7.53 ± 0.74^a^	7.07 ± 0.60^b^
Lime juice	6.27 ± 1.10^b^	6.27 ± 1.49^b^	6.87 ± 1.41^b^	6.27 ± 1.30^c^	6.73 ± 1.16^c^	6.40 ± 0.98^c^
Vinegar	6.93 ± 1.22^b^	7.07 ± 1.16^a^	7.53 ± 0.99^a^	7.13 ± 0.83^a^	7.07 ± 0.88^b^	7.27 ± 0.70^a^
Mean	6.97	6.97	7.08	6.95	7.35	7.15
*p* level	^**^	^**^	^**^	^**^	^**^	^**^

Values are the mean and ±standard deviation of duplicate determinations. Data with different superscripts within the same column are significantly different (p < 0.05). 9-like extremely, 8-like very much, 7-like moderately, 6-like slightly, 5-neither like nor dislike, 4-dislikes slightly, 3-dislikes moderately, 2-dislikes very much, 1-dislike extremely.

^**^p < 0.01.

The steeped *ogi* water-coagulated *Tofu* was the most liked in all the sensory attributes, including the overall acceptability. However, the steeped *ogi* water-coagulated *Tofu* is comparable to the vinegar-coagulated *Tofu* in terms of taste, appearance, aroma, and overall acceptability. This is because there was no significant difference (*p* > 0.05) in these attributes between the steeped ogi water-coagulated *Tofu* and vinegar-coagulated *Tofu*. This suggests that steeped *ogi* water and vinegar not only enhanced the visual and aromatic appeal of the product but also contributed positively to the flavor and mouthfeel of the *Tofu*. The mild fermentation imparted by the lactic acid in the steeped *ogi* water and the acetic acid in the vinegar may have helped develop deeper, more complex flavors and softened the texture, thereby improving the sensory acceptance (11).

Similarly, significant differences (*p* > 0.05) were not observed in the color and texture of the steeped ogi water-coagulated *Tofu* and alum-coagulated *Tofu*. Although the overall acceptability of the alum-coagulated *Tofu* was within a moderately liked range, the lower ratings in aroma and taste compared to those of the steeped ogi water-coagulated *Tofu* suggest that alum may have contributed more to the physical structure and color of the *Tofu* than to its flavor attributes.

As a result of its capacity to enhance flavor, fragrance, and overall sensory appeal, steeped *ogi* water was shown to be the most effective coagulant in improving the sensory aspects of *Tofu* and followed by vinegar, which provides a tasty and aesthetically pleasing product, while alum has less impact on taste and aroma. Lime was the least popular choice, perhaps because of its strong acidity and limited impact on positive sensory development.

### 3.6 Relationships between the chemical composition, textural attributes and sensory acceptability of *Tofu* from different coagulants

**Figure 1:**
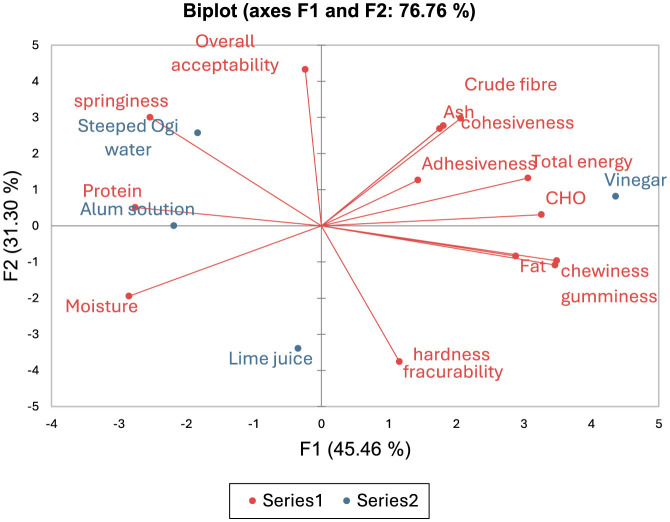
Principal component analysis biplot of the chemical composition, textural attributes and overall acceptability of the *Tofu* samples from different coagulants.

The principal component analysis (PCA) biplot (Figure 1) allowed us to draw a line between chemical composition, textural attributes and overall acceptability of the *Tofu* samples from different coagulants. The result showed a data variance of about 76.76%, with the principal component one (PC1) contributing 45.46% and principal component two (PC2) contributing 31.30% (Figure 1). The PC1 was positively correlated with chemical composition, such as fat (*r* = 0.79), crude fiber (*r* = 0.57), carbohydrate (*r* = 0.90), and total energy (*r* = 0.84), but negatively correlated with moisture (*r* = −0.78) and protein (*r* = −0.76). Also, the PC1 was positively correlated with texture attributes such as chewiness (*r* = 0.96) and gumminess (*r* = 0.95) (Supplementary Table 1). In addition, the PC2 had a positive correlation with the ash (*r* = 0.61) and crude fiber (*r* = 0.68) contents, cohesiveness (*r* = 0.63), springiness (*r* = 0.69), and overall acceptability (*r* = 0.99). The relationship between the PC2 and the fracturability (*r* = −0.86) and hardness (*r* = −0.86) was negative (Supplementary Table 1). The crude fiber, ash, carbohydrate and total energy contents of the vinegar-coagulated *Tofu* may have influenced its cohesiveness and adhesiveness as they all belong to the same quadrant (Figure 1). Similarly, the protein content of the alum- and steeped ogi water-coagulated *Tofu* may have influenced their springiness, and subsequent overall acceptability (Figure 1).

**Figure 2:**
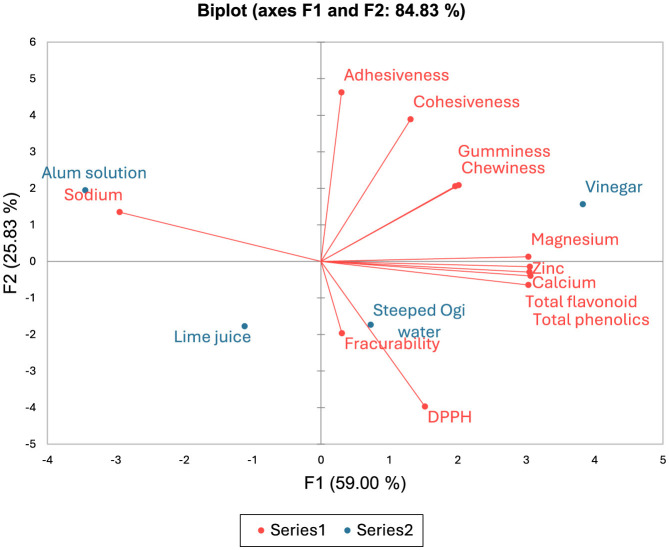
Principal component analysis biplot of the mineral composition, textural attributes and antioxidant properties of the *Tofu* samples from different coagulants.

Figure 2 shows the PCA biplot of mineral composition, textural attributes and antioxidant properties of the *Tofu* samples from different coagulants. A data variance of about 84.83% was shown in the result, with PC1 contributing 59% and PC2 contributing 25.83%. A positive correlation exists between PC1 and the calcium (*r* = 0.99), magnesium (*r* = 0.98), zinc (*r* = 0.99), total phenolics (0.98), total flavonoids (*r* = 1), chewiness (*r* = 0.65), and gumminess (*r* = 0.64). The correlation between sodium content and PC1 was negative (*r* = −0.96). The PC2 had a negative correlation with the DPPH (*r* = −0.85), and a positive correlation with the adhesiveness (*r* = 0.99) and cohesiveness (*r* = 0.84) (Supplementary Table 2). The adhesiveness, cohesiveness, gumminess, and chewiness of the vinegar-coagulated *Tofu* may have been influenced by its magnesium content because they all belong to the same quadrant in the PCA biplot. Also, the antioxidant activities (total flavonoids, total phenolics, and DPPH) of the steeped ogi water-coagulated *Tofu* may have influenced its zinc and calcium contents, and its subsequent fracturability (Figure 2)

## 4 Conclusion

The results of this study showed that vinegar-coagulated *Tofu* significantly possesses the highest fat, ash, crude fiber, total carbohydrate, and total energy contents, and the steeped Ogi water-coagulated *Tofu* had the highest protein content. The calcium, magnesium, and zinc contents were higher in the vinegar-coagulated *Tofu*, while the sodium content was higher in the alum solution-coagulated Tofu. Total phenolics and total flavonoids were higher in the vinegar-coagulated *Tofu*, while the steeped Ogi water-coagulated *Tofu* had the highest DPPH value. The lime-coagulated *Tofu* had the lowest of all the chemical compositions. The adhesiveness, chewiness, cohesiveness, and gumminess were higher in the vinegar-coagulated *Tofu*, while the fracturability and hardness were higher in the lime juice-coagulated *Tofu*. The crude fiber, ash, carbohydrate and total energy contents of the vinegar-coagulated *Tofu* may have influenced its cohesiveness and adhesiveness as they all belong to the same quadrant. The adhesiveness, cohesiveness, gumminess, and chewiness of the vinegar-coagulated *Tofu* may have been influenced by its magnesium content because they all belong to the same quadrant in the PCA biplot. Also, the antioxidant activities (total flavonoids, total phenolics, and DPPH) of the steeped ogi water-coagulated *Tofu* may have influenced its zinc and calcium contents, and its subsequent fracturability. Similarly, the protein content of the alum- and steeped ogi water-coagulated *Tofu* may have influenced their springiness, and subsequent overall acceptability. Therefore, steeped *Ogi* water coagulant could be used to produce quality *Tofu* that will balance the chemical composition, textural attributes and sensory acceptability. However, there is a need to evaluate the amino acid profiling of the steeped *Ogi* water coagulated *Tofu* and its consumer acceptability before commercialization.

## Data Availability

The original contributions presented in the study are included in the article/Supplementary material, further inquiries can be directed to the corresponding author/s.
