# Supplementary material for: Relationship between the chemical composition, textural attributes, and sensory acceptability of Tofu as influenced by different coagulants
Source: Front Nutr. 2025 Dec 17;12:1724587. doi: 10.3389/fnut.2025.1724587 (PMC12753399; doi:10.3389/fnut.2025.1724587)
Supplement: Supplementary file 1 [file Table_1.docx]

Supplementary Table 1. Correlation loading of the chemical composition, textural attributes, and overall acceptability of *Tofu* from different coagulants

| Attributes | F1 | F2 |
| --- | --- | --- |
| Moisture content | -0.78 | -0.44 |
| Protein content | -0.76 | 0.12 |
| Fat content | 0.79 | -0.19 |
| Ash content | 0.48 | 0.61 |
| Crude fibre content | 0.57 | 0.68 |
| Carbohydrate content | 0.90 | 0.07 |
| Total energy content | 0.84 | 0.30 |
| Adhesiveness | 0.39 | 0.29 |
| Chewiness | 0.96 | -0.22 |
| Cohesiveness | 0.50 | 0.63 |
| Fracturability | 0.32 | -0.86 |
| Gumminess | 0.95 | -0.25 |
| Hardness | 0.32 | -0.86 |
| Springiness | -0.70 | 0.69 |
| Overall acceptability | -0.07 | 0.99 |

Supplementary Table 2. Correlation loading of the mineral composition, textural attributes and antioxidant properties of the *Tofu* samples from different coagulants

| Attributes | F1 | F2 |
| --- | --- | --- |
| Calcium | 0.99 | -0.06 |
| Magnesium | 0.98 | 0.03 |
| Zinc | 0.99 | -0.03 |
| Sodium | -0.96 | 0.29 |
| Total phenolics | 0.98 | -0.14 |
| Total flavonoid | 0.99 | -0.09 |
| DPPH | 0.49 | -0.85 |
| Adhesiveness | 0.10 | 0.99 |
| Chewiness | 0.65 | 0.45 |
| Cohesiveness | 0.43 | 0.84 |
| Fracturability | 0.10 | -0.42 |
| Gumminess | 0.64 | 0.44 |
